# Alternative Complement Activity in the Egg Cytosol of Amphioxus *Branchiostoma belcheri*: Evidence for the Defense Role of Maternal Complement Components

**DOI:** 10.1371/journal.pone.0004234

**Published:** 2009-01-21

**Authors:** Yujun Liang, Shicui Zhang, Zhiping Wang

**Affiliations:** 1 Department of Marine Biology, Ocean University of China, Qingdao, People's Republic of China; 2 Ocean College of Hebei Agricultural University, Qinhuangdao, People's Republic of China; University of Birmingham, United Kingdom

## Abstract

**Background:**

The eggs in most invertebrates are fertilized externally, and therefore their resulting embryos are exposed to an environment full of microbes, many of which are pathogens capable of killing other organisms. How the developing embryos of invertebrates defend themselves against pathogenic attacks is an intriguing question to biologists, and remains largely unknown.

**Methodology/Principal Findings:**

Here we clearly demonstrated that the egg cytosol prepared from the newly fertilized eggs of amphioxus Branchiostoma belcheri, an invertebrate chordate, was able to inhibit the growth of both the Gram-negative bacterium Vibrio anguillarum and the Gram-positive bacterium Staphylococcus aureus. All findings point to that it is the complement system operating via the alternative pathway that is attributable to the bacteriostatic activity.

**Conclusions/Significance:**

This appears to be the first report providing the evidence for the functional role of the maternal complement components in the eggs of invertebrate species, paving the way for the study of maternal immunity in other invertebrate organisms whose eggs are fertilized in vitro. It also supports the notion that the early developing embryos share some defense mechanisms common with the adult species.

## Introduction

Invertebrates hold a dominant position in the kingdom Animalia. It is estimated that the described species of invertebrates is 1.325 million, accounting for about 97% of the total animal species [1]. The eggs in most invertebrates are fertilized externally, and therefore their resulting embryos are exposed to an environment full of microbes, many of which are pathogens capable of killing other organisms. It is conceivable that the embryos have the ability to defend themselves against pathogens and protect themselves from diseases. However, little information as such is available in the huge number of invertebrates, and how they survive pathogenic attacks remains largely unknown.

Complement is a major effector system of innate immunity, which plays an essential role in alerting the host immune system of the presence of potential pathogens as well as their clearance. Complement system consisting of approximately 35 plasma and membrane-bound proteins can be activated by three different but partially overlapping routes: the classical pathway (CP), the alternative pathway (AP) and the lectin pathway (LP). The CP activation is initiated by binding of antibody to the C1 complex, formed by C1q and two serine proteases (C1r and C1s), or by direct binding of the C1q component to the pathogen surface, and requires both Ca^2+^ and Mg^2+^ [2]–[4]. The AP is mainly triggered by the certain structures on microbial surface in an antibody-independent manner, and requires Mg^2+^ alone [5]. The C3 is cleaved spontaneously in plasma to yield C3b which interacts non-covalently with factor B (Bf) and factor D, resulting in the formation of the alternative C3 convertase [6]. The LP is activated by binding of microbial polysaccharides to circulating lectins, such as mannose-binding lectin (MBL), and requires Ca^2+^ [7]–[10]. MBL binds to the mannose residues which then results in the cleavage of C4 via mannose-binding protein-associated serine esterase. All the three pathways merge at a common amplification step involving C3, a central complement component being a part of all the three pathways, and proceed through a terminal pathway that leads to the formation of a membrane attack complex, which can directly lyse pathogenic cells. Maternal transfer of complement components such as C3 and Bf to offspring has recently been demonstrated in several fishes including rainbow trout [11] and zebrafish [12]. Moreover, these maternally-transferred molecules have been shown to be involved in the defense of piscine embryos against pathogens [12]. Likewise, maternal transmission of immunity has also been demonstrated in several invertebrates such as bumblebees [13] and crustacean [14], [15], yet the nature of the transferred factors is elusive.

Complement, as an ancient defense system, can be traced from the coral and sea anemone, members of the protostome *Cnidaria* [16], [17], and is widely present in the invertebrates including horseshoe crab [18], sea urchin [19] and sea squirt [20]. It has been shown that sea urchin, in which two components with significant homology to vertebrate C3 and Bf have been identified, possesses a simplified system homologous to the AP [21], [22]. Both C3-like and C6-like molecules as well as Bf have also been documented in the basal chordate amphioxus *Branchisotoma belcheri* [23]–[25], and genome analysis revealed the presence of hundreds of molecules containing complement-related domains, implying the existence of a complicated complement system in amphioxus *B. floridae* [26]. In addition, a complement system operating via the AP similar to that of vertebrates has been shown to function in the adults of *B. belcheri* [27], [28]. The aims of this study were therefore to determine if the key components C3 and Bf involved in the AP are present in the eggs of amphioxus *B. belcheri*, and if so, to examine if they are associated with the immune defense of early developing embryos.

## Results

### Bacteriostatic activity of egg cytosol

**Figure 1:**
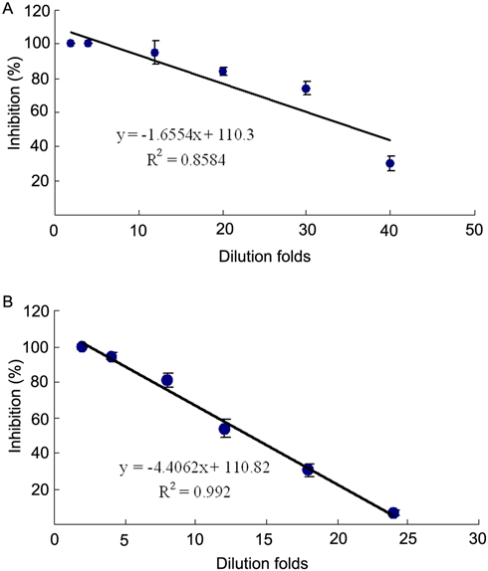
Bacteriostatic activity in amphioxus egg cytosol. The egg cytosol was diluted at different folds and mixed with *Vibrio anguillarum* (A) and *Staphylococcus aureus* (B) suspensions, respectivly. The mixtures were incubated at 25°C for 1 h, and the bacteriostatic activity was determined by colony forming unit assay. A regression was done to show the differences between the control and tests. R means the correlation coefficient.

The protein concentrations of the egg cytosols prepared from the fertilized eggs of *B. belcheri* ranged from 16.5 mg/ml to 12.6 mg/ml, with an average of 14.0 mg/ml. The egg cytosol demonstrated a conspicuous bacteriostatic activity against the Gram-negative bacterium *Virio anguillarum* as well as the Gram-positive bacterium *Staphylococcus aureus* (Figure 1A and B). The bacteriostatic activity against both *V. anguillarum* and *S. aureus* was in a dose-dependent manner. When the egg cytosol was diluted 2-, 4-, 12-, 20-, 30-, 40- and 50-folds, the inhibition rates against *V. anguillarum* were 100%, 100%, 95±8.1%, 84±2.2%, 74±4.1%, 30±4.0% and 10±2.5%, respectively. Similarly, when the cytosol was diluted 2-, 4-, 8-, 12-, 18- and 24-folds, the inhibition rates against *S. aureus* were 100%, 94±2.5%, 81±3.9%, 54±3.7%, 30±3.7% and 6±1.8%, individually.

### Complement activity

**Figure 2:**
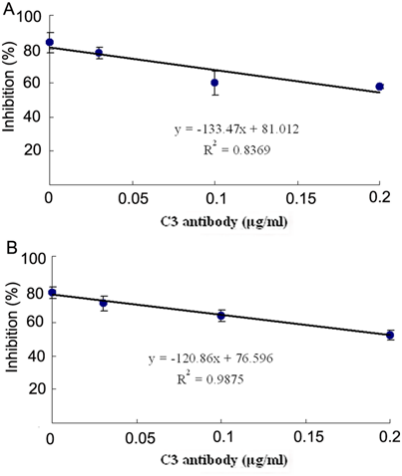
Effects of anti-C3 antibody on the bacteriostatic activity of amphioxus egg cytosol. The egg cytosol was pre-incubated with anti-C3 antibody at different concentrations before the addition of *Vibrio anguillarum* (A) and *Staphylococcus aureus* (B) suspensions, respectively. The mixtures were incubated at 25°C for 1 h, and the bacteriostatic activity was determined by colony forming unit assay. A regression was done to show the differences between the control and tests. R means the correlation coefficient.

**Figure 3:**
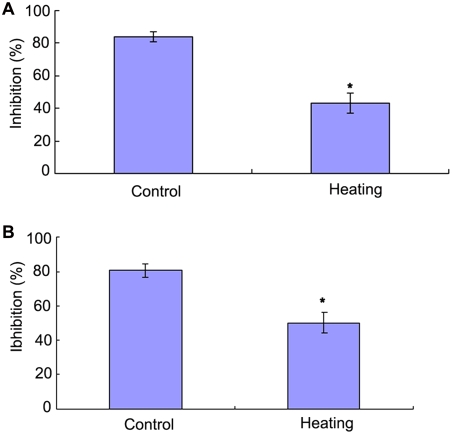
Effects of heating on the bacteriostatic activity of amphioxus egg cytosol. The egg cytosol was inactivated by heating at 45°C for 30 min before the addition of *Vibrio anguillarum* (A) and *Staphylococcus aureus* (B) suspensions, respectively. The mixtures were incubated at 25°C for 1 h, and the bacteriostatic activity was determined by colony forming unit assay. The symbol * means *p*<0.05.

Pre-incubation of anti-C3 antibody with the egg cytosol was capable of reducing the bacteriostatic activity against both *V. anguillarum* and *S. aureus* in a concentration-dependent fashion (Figure 2A and B), strongly suggesting a role for complement in the bacteriostatic activity of the egg cytosol. This was further strengthened by the fact that heating the egg cytosol (45°C, 30 min) significantly reduced (*p*<0.01) the bacteriostatic activity against *V. anguillarum* and *S. aureus*, with the inhibition rates of 43±6.0% and 50±6.2% observed, contrasting to those of 84±3.1% and 81±4.2% in control (Figure 3A and B).

**Figure 4:**
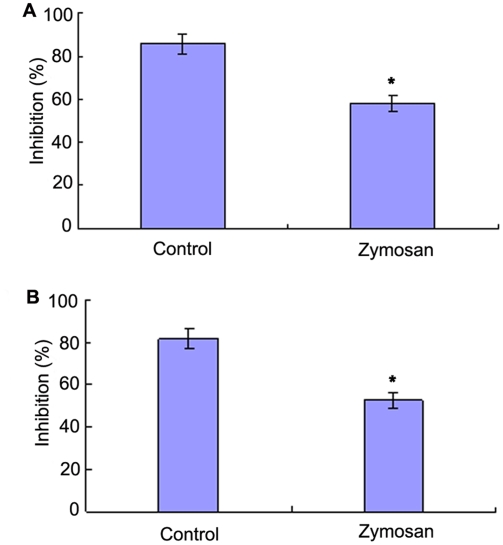
Effects of zymosan on the bateriostatic activity of amphioxus egg cytosol. The egg cytosol was pre-incubated with zymason, and mixed with *Vibrio anguillarum* (A) and *Staphylococcus aureus* (B) suspensions, respectively. The mixtures were incubated at 25°C for 1 h, and the bacteriostatic activity was determined by colony forming unit assay. The symbol * means *p*<0.05.

**Figure 5:**
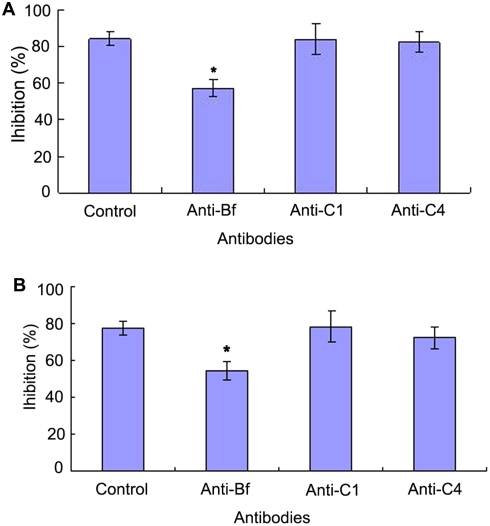
Effects of complement antibodies on the bateriostatic activity of amphioxus egg cytosol. The egg cytosol was pre-incubated with complement antibodies against Bf, C1q and C4 at optimal concentrations, and mixed with *Vibrio anguillarum* (A) and *Staphylococcus aureus* (B) suspensions, respectively. The mixtures were incubated at 25°C for 1 h, and the bacteriostatic activity was determined by colony forming unit assay. The symbol * means *p*<0.05.

**Figure 6:**
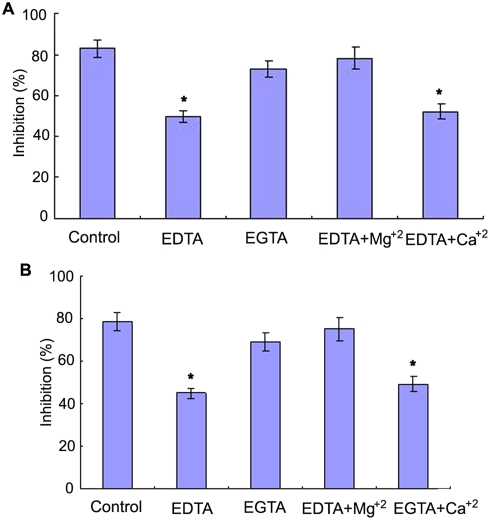
Effects of divalent cation chelators EGTA and EGTA on the bacteriostatic activity of amphioxus egg cytosol. The egg cytosol was pre-incubated with EGTA, EDTA, EDTA with Mg^2+^ and EDTA with Ca^2+^ at optimal concentrations, and mixed with *Vibrio anguillarum* (A) and *Staphylococcus aureus* (B) suspensions, respectively. The mixtures were incubated at 25°C for 1 h, and the bacteriostatic activity was determined by colony forming unit assay. The symbol * means *p*<0.05.

C3b has been shown to be able to covalently bind to zymosan particles [30] and thus the AP-mediated bacteriostatic activity can be selectively inhibited by addition of zymosan. It was found that the bacteriostatic activities of the egg cytosol against *V. anguillarum* and *S. aureus* were significantly decreased by pre-incubation with zymosan, with the inhibition rates of 58±3.8% and 52±4.0% contrasting to those of 86±4.6% and 82±4.9% in control (Figure 4A and B). Similarly, pre-incubation with the antibody against Bf, a key enzyme in the AP activation, also resulted in a remarkable decrease in the bacteriostatic activities of the egg cytosol against *V. anguillarum* and *S. aureus*, with the inhibition rates of 57±4.8% and 55±5.0% contrasting to those of 85±3.7% and 77±3.7% in control (Figure 5A and B). In contrast, pre-incubation with either anti-C1q antibody or anti-C4 antibody had little effects on the bacteriostatic activities against *V. anguillarum* and *S. aureus* (Figure 4A and B). All these suggested that the activation of the AP was contributable to the bacteriostatic activity. In agreement, addition of 0.45 mM EGTA to the egg cytosol did not impair the bacteriostatic activities against *V. anguillarum* and *S. aureus*, while addition of 0.45 mM EDTA to the cytosol significantly reduced its bacteriostatic activities (Figure 5A and B). Moreover, the bacteriostatic activities of the EDTA-treated egg cytosols were capable of being rescued by addition of Mg^2+^, but not by addition of Ca^2+^ (Figure 6A and B).

### Presence of C3 and Bf in egg cytosol

**Figure 7:**
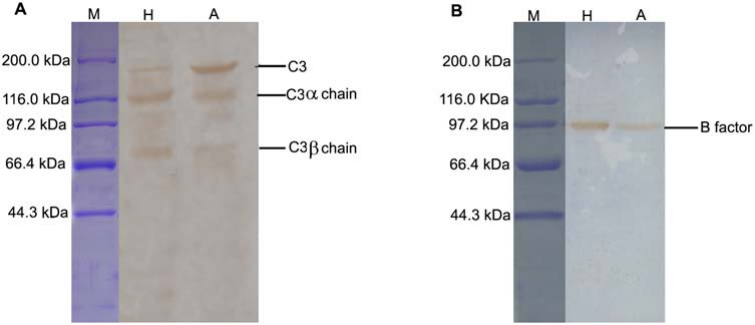
Western blotting of C3 (A) and Bf (B) in amphioxus egg cytosol. Lane M, standard marker; Lane H, human serum (control); Lane A, amphioxus egg cytosol.

Western blotting showed that anti-C3 antibody reacted with the egg cytosol as well as with human serum. The egg cytosol was reactive with rabbit anti-human C3 antibody, forming a main band (∼180 kDa) equivalent to C3 and two minor bands (∼110 kDa; ∼70 kDa) resembling C3*α* and C3β chains, respectively (Figure 7A). Similarly, the egg cytosol also reacted with goat anti-human Bf antibody, producing a single positive band of about 93 kDa, matching that of human Bf (Figure 7B).

It was revealed by ELISA that the concentrations of C3 and Bf in the egg cytosol prepared were about 318 µg/ml and 46 µg/ml, respectively. In contrast, the contents of C3 and Bf in each egg of *B. belcheri* were deduced to be approximately 5483 µg/ml and 833 µg/ml, that were both about 17 times higher than those in the egg cytosol.

## Discussion

The existence of complement components like C3 and Bf has recently been proved in the eggs of several fishes including zebrafish, rainbow trout, carp and spotted wolfish. In this study we clearly demonstrate the presence of both C3 and Bf, two key factors functioning in the AP, in the newly fertilized eggs of the invertebrate chordate *B. belcheri*, providing the first evidence for a maternal transfer of the complement proteins in this evolutionarily important animal.

The role of maternal complement components in fish eggs has been proposed to be involved in the early defense against pathogenic microbes in the developing embryos. However, the functional studies as such remain largely lacking, and little is known about the protection of early embryos against microbial attack in the invertebrates. Here we showed for the first time that the growth of the Gram-negative bacterium *V. anguillarum* and the Gram-positive bacterium *S. aureus* are readily inhibited by the egg cytosol prepared from the fertilized eggs of amphioxus *B. belcheri*, and all the findings point to the complement system being one of the most crucial factors involved in the bacteriostatic activities observed. First, the bacteriostatic activities were reduced by pre-incubation of anti-C3 antibody with the egg cytosol, a process that would cause the precipitation of the central component of all known complement pathways, C3. Second, the bacteriostatic activities were decreased by heating at 45°C, a temperature known to inactivate amphioxus complement [28], [29]. It is highly likely that as with fish eggs, the maternal complement components may also function in the early embryos, protecting the developing amphioxus embryos from microbial attacks. In sea urchin, C3 in the coelomic fluid has been shown to function as an opsonin and opsonnizes yeast (*Saccharomyces cerevisiae*) to be phagocytosed by the coelomocytes, polygonal phagocytes [21], whether C3 in *B. belcheri* can act similarly demands further study.

To determine which pathway of the complement activation might be associated with the bacteriostatic activity of the egg cytosol, the antibodies against C1q (a key component of CP), C4 (a key component of both CP and LP) and Bf (a key component of AP) were utilized to block the CP, LP or AP, respectively. It is found that the precipitation of C1q and C4 results in little loss of the bacteriostatic activity of the egg cytosol, whereas the precipitation of Bf leads to a significant reduction of the bacteriostatic activity. Furthermore, the addition of EGTA to remove Ca^2+^ from the egg cytosol, which can inhibit both CP and LP, induces little decrease in the bacteriostatic activity. In contrast, the pre-incubation of EDTA with the egg cytosol causes a substantial reduction of the bacteriostatic activity, and the saturation of the chelator with Mg^2+^ is able to rescue the bacteriostatic activity, but not by the addition of Ca^2+^. Moreover, the selective inhibition of the AP by zymosan triggers a marked loss of bacteriostatic activity. Taken together, all these undoubtedly indicate that the activation of the AP is primarily responsible for the bacteriostatic activity of the egg cytosol, but the CP and LP have little contribution to the bacteriostatic activity. It is also interesting to note that the egg of *B. belcheri* contains abundant C3 and Bf, making the early embryo an effective system coping with potential pathogens.

In summary, the present study proves that the complement components C3 and Bf can be maternally transferred to offspring in the invertebrate chordate amphioxus, and the complement system operating via the AP can play an important role in the protection of the developing embryos against pathogenic attack. This appears the first report highlighting the functional role of complement system in the embryogenesis of invertebrate species. It also bolsters the notion that the early developing embryos share some defense mechanisms common with the adult species.

## Materials and Methods

### Chemicals

Ethylenediamine tetraacetic acid (EDTA), ethyleneglycol-bis (β-aminoethyl ether)-N,N,N′,N′-tetraacetic acid (EGTA), o-phenylenediamine, zymosan A, and bovine serum albumin (BSA) were purchased from Sigma (St. Louis, USA), and peptone and yeast extract were from OXOID (Basingstoke, UK). Rabbit anti-human C3 antibody was procured from Abcam (Cambridge, UK), goat anti-human factor B (Bf) antibody from R & D system (Minneapolis, USA), goat anti-human C1q antibody and rabbit anti-mouse C4 antibody from Boster (Wuhan, China), and horseradish peroxidase (HRP)-conjugated rabbit anti-goat IgG and HRP-labeled goat anti-rabbit IgG from Jackson (Baltimore, USA). BCA protein assay Kit was from Beyotime (Haimen, China), ELISA assay kit for C3 and ELISA assay kit for B factor were from ADLITTERAM disgnostic laboratories (Fremont, USA). All other chemicals used were analytical reagents.

### Preparation of the egg cytosol

The naturally fertilized eggs were collected at 1- to 4-cell stage, washed three times in sterilized seawater and once in sterilized PBS, and homogenized on ice for 40 seconds. After centrifugation at 15 000 g at 4°C for 30 min, the supernatants, egg cytosols, were pooled, aliquoted and stored at −80°C until used.

The protein concentrations were determined using BCA protein assay kit.

### Preparation of bacteria

The Gram-negative bacterium *Vibrio anguillarum* and Gram-positive bacterium *Staphylococcus aureus* were both cultured in 2611E medium to logarithmic growth phase, and harvested by centrifugation at 4 000 g for 5 min. The pellets were washed three times with sterilized 20 mM phosphate buffered saline (PBS; pH 7.4), re-suspended at a density of 10^5^ cells/ml, and used for the following experiments.

### Assays for bacteriostatic activity

The egg cytosol was filtered through 0.22 µm filter (Millipore) before use. An aliquot of 100 µl of the egg cytosols, that were diluted at different folds with sterilized PBS, was mixed with 20 µl of *V. anguillarum* and *S. aureus* suspensions (10^5^ cells/ml), respectively, and the mixtures were pre-incubated, with gentle stirring, at 25°C for 1 h. Subsequently, the mixtures were plated onto 3 LB agar plates (30 µl each plate). After incubation at 28°C for 16 h, the resulting bacterial colonies in each plate were counted. The control was processed similarly except that the egg cytosol was replaced with sterilized 20 mM PBS (pH 7.4). The percent of bacterial growth inhibition by the egg cytosol was inferred from the difference between the numbers of colonies in the test and control.

### Assays for inhibition of complement

In the following experiments using *V. anguillarum* and *S. aureus*, the egg cytosol was diluted with PBS 20- and 8-folds, respectively. The capacity of the antibodies against C3, Bf, C1q and C4 to inhibit the bacteriostatic activity of the egg cytosol was analyzed according to the method of Wang et al. [12]. In brief, the egg cytosol was pre-incubated with either anti-C3 antibody at concentrations of 0.03, 0.1 and 0.20 µg/ml or anti-C1q, C4 and Bf antibodies at final concentrations of 0.01, 0.08 and 0.24 µg/ml at 25°C for 30 min, followed by addition of 20 µl of *V. anguillarum* and *S. aureus* suspensions (10^5^ cells/ml), respectively. The mixtures were adjusted to a volume of 120 µl with 20 mM PBS (pH 7.4), and incubated at 25°C for 1 h. The bacteriostatic activities were measured as described above. For control, the antibodies were replaced by sterilized PBS.

Aliquots of the egg cytosol diluted were inactivated by heating at 45°C for 30 min [29], [31], and mixed with 20 µl of *V. anguillarum* and *S. aureus* suspensions (10^5^ cells/ml), respectively. The mixtures were adjusted to 120 µl with 20 mM PBS (pH 7.4), incubated at 25°C for 1 h, and their remaining bacteriostatic activities assayed. The egg cytosol without heating was used as control.

For chelation experiments, 6 µl of the egg cytosol was mixed with 4.5 µl of 10 mM EGTA, EDTA, EDTA with MgCl_2_ and EDTA with CaCl_2_, respectively, and adjusted to 100 µl with sterilized PBS. After pre-incubation at 25°C for 30 min, the mixtures were combined with 20 µl of *V. anguillarum* suspension (10^5^ cells/ml). After incubation at 25°C for 30 min, the bacteriostatic activity was determined. Similarly, 15 µl of the egg cytosol was mixed with 4.5 µl of 10 mM EGTA, EDTA, EDTA with MgCl_2_ and EDTA with CaCl_2_, respectively, adjusted to 100 µl with sterilized PBS, and pre-incubated; after combination with 20 µl of *S. aureus* suspension (10^5^ cells/ml), the bacteriostatic activity was detected. Control was processed similarly except that the chelator solutions were replaced by sterilized PBS.

The stock solution of 10 mg/ml zymosan A was prepared by boiling 10 mg zymosan A in 1 ml of 14 mM NaCl for 30 min. To inhibit the alternative pathway, 100 µl (1 mg of zymosan) of the stock solution was centrifuged at 16 000 g for 5 min, and the zymosan pellet was re-suspended in 100 µl of the egg cytosol diluted. After pre-incubation at 25°C for 30 min, the zymosan in the reaction medium was removed by centrifugation at 16 000 g for 5 min. The resulting egg cytosol was mixed with 20 µl of *V. anguillarum* and *S. aureus* suspensions (10^5^ cells/ml), respectively. The mixture was incubated at 25°C for 1 h, and the bacteriostatic activity was tested.

### SDS-PAGE and Western blotting

The egg cytosol was run on a 10% SDS-PAGE gel with a 4% spacer gel using the buffer system of Laemmli [32]. Human serum was also run on the gel as a positive control at the same time. The gels were washed for 5 min in 15.6 mM Tris-HCl (pH 8.3) containing 120 mM glycine and 20% methanol, and the proteins on the gels were blotted on a nitrocellulose membrane (Hybond, Amersham Pharmacia). The blotted membranes were incubated in 20 mM PBS (pH 7.4) with 50 mM NaCl and 5% defatted milk powder at room temperature for 1 h, washed three times with 20 m PBS containing 50 m NaCl, and then reacted with the rabbit anti-human C3 antibody diluted 1∶1200 and goat anti-human Bf antibody diluted 1∶300 at room temperature for 1.5 h. After washing with 20 mM PBS, the membranes were incubated with peroxidase-conjugated goat anti-rabbit IgG and rabbit anti-goat IgG that were both diluted 1∶1000 at room temperature for 50 min. The bands were visualized using 0.06% DAB in 50 mM Tris-HCl (pH 8.3). The molecular markers used were myosin (200 kDa), β-galactosidase (116 kDa), phosphorylase B (97.2 kDa), serum albumin (66.4 kDa) and ovalbumin (44.3 kDa).

### Titration of C3 and Bf in egg cytosol and each egg

ELISA kit was used to determine the concentrations of C3 and Bf in the egg cytosol prepared from 67.5×10^3^ eggs. The wells in a 96-well polystyrene plate were coated with standard C3 and Bf solutions, and the egg cytosol, and placed at 4°C overnight. The wells were washed five times with the washing buffer and blocked with PBST-BSA buffer (2% BSA and 0.1% Tween-20 in PBS, pH 7.4). After washing, the anti-C3 antibody (diluted 1∶2000) and anti-Bf antibody (diluted 1∶800) were added to the wells, respectively, and incubated at room temperature for 1 h. Subsequently, they were washed five times, and incubated with HRP-labeled anti-goat and anti-rabbit IGg antibodies at 37°C for 1 h. The coloration was carried out in dark at 37°C for 15 min, and terminated with the stopping solution (200 mM H_2_SO_4_). The absorbance (optical density) was measured at 492 nm under a microplate reader (TECAN Genios Plus, Austria). The concentrations of C3 and Bf in the egg cytosol were calculated using the standard curve. The mean diameter of *B. belcheri* eggs was 140 µm [33], and therefore the egg volume was approximately 1.44×10^−6^ cm^3^. Accordingly, the contents of C3 and Bf in each egg were deduced.

### Statistical analysis

All experiments were performed in triplicate, and repeated at least three times. Data were expressed as a mean±standard deviation (SD). Data were subjected to statistical evaluation with ANOVA, and difference at *p*<0.05 was considered significant. To the continuous variable, a regression was done to show the differences between the control and tests.
